# Draft Genome Sequences of the Three Pectobacterium-Antagonistic Bacteria Pseudomonas brassicacearum PP1-210F and PA1G7 and Bacillus simplex BA2H3

**DOI:** 10.1128/genomeA.01497-14

**Published:** 2015-01-29

**Authors:** Slimane Khayi, Yannick Raoul des Essarts, Samuel Mondy, Mohieddine Moumni, Valérie Hélias, Amélie Beury-Cirou, Denis Faure

**Affiliations:** a CNRS, Institut des Sciences du Végétal, UPR2355, Saclay Plant Sciences, Gif-sur-Yvette, France; b Faculté des Sciences, Département de Biologie, Université Moulay Ismail, Meknès, Morocco; c Fédération Nationale des Producteurs de Plants de Pomme de Terre-Recherche Développement Promotion du Plant de Pomme de Terre (FN3PT-RD3PT), Paris, France; d Comité Nord Plant de Pomme de Terre (CNPPT), Semences, Innovation, Protection Recherche et Environnement (SIPRE), Achicourt, France; e UMT Innoplant (FN3PT-INRAIGEPP1349), Le Rheu, France

## Abstract

Pectobacterium spp. are bacterial pathogens causing soft rot diseases on a wide range of plants and crops. We present in this paper the draft genome sequences of three bacterial strains, Pseudomonas brassicacearum PP1-210F and PA1G7 and Bacillus simplex BA2H3, which exhibit antagonistic activities against the Pectobacterium plant pathogens.

## GENOME ANNOUNCEMENT

Pectobacterium atrosepticum, Pectobacterium wasabiae, and Pectobacterium carotovorum, including P. carotovorum subsp. brasiliensis and carotovorum, are worldwide pathogens responsible for blackleg and soft rot diseases on potato plants and tubers (1–3). Two biocontrol strategies against Pectobacterium phytopathogens have been developed, those of antibiosis and antivirulence (4). The biocontrol strain Rhodococcus erythropolis R138 targets the expression of the virulence functions in Pectobacterium spp. because of its capacity to disrupt the quorum-sensing signals *N*-acylhomoserine lactones (5). The genome sequence of the R. erythropolis antivirulence agent R138 was published recently (6). This antivirulence agent does not inhibit the growth of Pectobacterium spp. In contrast, we isolated three Pectobacterium-antagonistic bacteria, Pseudomonas brassicacearum PP1-210F and PA1G7 and Bacillus simplex BA2H3, which exhibit an ability to inhibit the growth of Pectobacterium strains *in vitro*. An assessment of their antagonistic abilities in the greenhouse and field settings is under way. Some other strains belonging to the Pseudomonas fluorescens, P. brassicacearum, and B. simplex species were previously described for their biocontrol activities against different microbial pathogens (7–10).

The genomic DNA of each bacterium was subjected to the next-generation Illumina HiSeq 2000 version 3 technology. A shotgun long jumping distance mate-pair library was constructed, with an insert size of 8,000 bp. The sequencing of the library was carried out using a 2 by 100-bp paired-end read module by Eurofins Genomics (France). Assembly was performed by CLC Genomics 5.5 (CLC bio). The sequence reads were trimmed on quality (threshold 0.05), and minimal size (>60 nucleotides). Contigs were generated by *de novo* assembly (CLC parameters, automatic determination of the word and bubble sizes with no scaffolding). Scaffolding of the contigs was performed using SSPACE basic version 2.0 (11). For the finishing, automatic gap closure was processed using GapFiller version 1.11 (12). The remaining gaps were resolved by the mapping of mate pairs, using as a reference the 8 kb from each of the contig ends (read length, 0.9; identity, 0.95). Next, using homemade script and fastq select.tcl from the MIRA3 package, the mapped reads for both orientations (R1 and R2) were retrieved and *de novo* assembled (using the CLC parameters). The sequences were annotated using the Rapid Annotations using Subsystems Technology (RAST) pipeline (13). The detailed statistics for the three draft genome sequences are summarized in Table 1.

**TABLE 1:** Statistics for the 3 draft genome sequences

Organism	Accession no.	Genome size (bp)	*N*_50_ (bp)	No. of contigs	No. of scaffolds	G+C content (%)	No. of CDSs^*a*^	No. of tRNAs	No. of rRNAs
B. simplex BA2H3	AXBR00000000	5,542,531	339,104	34	11	40.2	5,856	75	31
P. brassicacearum PP1-210F	AYJR00000000	6,772,045	210,148	51	5	60.4	6,045	67	15
P. brassicacearum PA1G7	JBON00000000	6,789,417	301,959	53	8	60.5	6,052	57	13

a CDSs, coding DNA sequences.

### Nucleotide sequence accession numbers.

The whole-genome shotgun projects for these bacteria have been deposited at DDBJ/EMBL/GenBank under the accession numbers AYJR00000000 (P. brassicacearum PP1-210F), AXBR00000000 (B. simplex BA2H3), and JBON00000000 (P. brassicacearum PA1G7). The versions described in this paper are versions AYJR01000000 (P. brassicacearum PP1-210F), AXBR01000000 (B. simplex BA2H3), and JBON01000000 (P. brassicacearum PA1G7).
